# COVID-19 vaccine: A 2021 analysis of perceptions on vaccine safety and promise in a U.S. sample

**DOI:** 10.1371/journal.pone.0268784

**Published:** 2022-05-19

**Authors:** Vitalis C. Osuji, Eric M. Galante, David Mischoulon, James E. Slaven, Gerardo Maupome

**Affiliations:** 1 Department of Global Health, Indiana University School of Medicine, Indianapolis, Indiana, United States of America; 2 Department of Psychiatry and Behavioral Sciences, Emory University School of Medicine, Atlanta, Georgia, United States of America; 3 Department of Psychiatry, Harvard Medical School, Boston, Massachusetts, United States of America; 4 Department of Biostatistics and Health Data Science, Indiana University School of Medicine, Indianapolis, Indiana, United States of America; 5 Department of Global Health, Indiana University Richard M. Fairbanks School of Public Health, Indianapolis, Indiana, United States of America; University of Texas Health Science Center at San Antonio, UNITED STATES

## Abstract

**Background:**

Despite reliable evidence-based research supporting the COVID-19 vaccines, population-wide confidence and trust remain limited. We sought to expand prior knowledge about COVID-19 vaccine perceptions, while determining which population groups are at greatest risk for not getting a vaccine.

**Methods:**

Study participants in the U.S. (79% female, median age group 46–60 years) were recruited through an online Qualtrics survey distributed as a Facebook advertisement from 3/19/21–4/30/21. We assumed that every participant is at risk of COVID-19 infection and should be able to get the vaccine with proper access. Bivariate and multivariable models were performed. Collinearity between variables was assessed.

**Results:**

A total of 2,626 responses were generated and 2,259 were included in data analysis. According to our multivariate model analysis, vaccines were perceived as safe by those who had or planned to obtain full vaccination (adjusted odds ratio (aOR) (95% confidence interval) = 40.0 (19.0, 84.2); p< 0.0001) and those who indicated trust in science (aOR = 10.5 (5.1, 21.8); p< 0.0001); vaccines were perceived as not safe by those who self-identified as Republicans vs. self-identified Democrats (aOR = 0.2 (0.1, 0.5); p = 0.0020) and those with high school or lower education (aOR = 0.2 (0.1, 0.4); p = 0.0007). Similarly, according to our multivariate model analysis, the following groups were most likely to reject vaccination based on belief in vaccinations: those with lower income (aOR = 0.8 (0.6, 0.9); p = 0.0106), those who do not know anyone who had been vaccinated (aOR = 0.1 (0.1, 0.4); p< 0.0001), those who are unwilling to get vaccinated even if family and friends had done so (aOR = 0.1 (<0.1, 0.2); p< 0.0001), those who did not trust science (aOR < 0.1 (<0.1, 0.1); p< 0.0001), those who believe that vaccination was unnecessary if others had already been vaccinated (aOR = 2.8 (1.5, 5.1); p = 0.0007), and those who indicate refusal to vaccinate to help others (aOR = 0.1 (0.1, 0.2); p< 0.0001). An alpha of p<0.05 was used for all tests.

**Conclusion:**

Level of education and partisanship, but not race/ethnicity, were the most likely factors associated with vaccine hesitancy or likelihood to vaccinate. Also, low vaccination rates among underrepresented minorities may be due to distrust for healthcare industries. Population sub-groups less likely to be vaccinated and/or receptive to vaccines should be targeted for vaccine education and incentives.

## Introduction

In early 2020, the SARS-CoV-2 (COVID-19) pandemic unmasked the many flaws that healthcare systems faced worldwide. While some of these issues were difficult to predict, such as the feasibility of pandemic response protocols or federal government regulations to be activated [1], other healthcare issues were to be expected, especially in the United States. For example, disparities in healthcare treatment and outcomes derived from different socioeconomic factors. Studies published in 2020 showed that the pandemic had much higher infection rates in minority populations such as Black and Hispanic/Latinx compared to their white counterparts; American Indians/ Alaska Natives (AI/ ANs), Black and Hispanic/Latinx communities also experienced significantly higher mortality rates [2, 3]. The Centers for Disease Control and Prevention (CDC) released information relating social determinants of health to poorer COVID-19 outcomes, stating that “factors such as discrimination, neighborhood and physical environment, housing, occupation, education, income, and wealth gaps put some racial and ethnic minority groups at increased risk of severe illness from COVID-19, including death” [4]. Many factors play a role in disparities relevant to the COVID-19 pandemic. These include limited access to health services, education, and transportation, which tend to affect more severely communities of color and people of low socioeconomic status [5].

Just under one year after the first identification of COVID-19 in China [6, 7], the PfizerBioNTech and Moderna COVID-19 vaccines were approved by the US Food and Drug Administration (FDA) under Emergency Use Authorization [8, 9]. Ultimately, the Pfizer vaccine was fully approved as of August 23^rd^, 2021. These vaccines represented a major milestone in vaccine production history, as no other vaccine had ever been created so rapidly with such positive results [10]. Although mistrust of vaccines is not uncommon in American culture, hesitation regarding the COVID-19 vaccines may be among the strongest yet [11]. Despite substantial evidence-based research supporting the vaccines’ safety and efficacy, there are lay public concerns regarding the vaccine rollout. For instance, an analysis [12] from March 2021 in individuals getting vaccines showed that white Americans were receiving vaccinations at a rate two times that of Black Americans, and the gap for Hispanic/Latinx was even larger. The rationale behind these gaps between racial/ethnic groups remains uncertain and highlights the importance of characterizing the factors and mechanisms underlying potential associations amongst demographic and socioeconomic groups.

With the current vaccines showing 95% efficacy, the estimated percentage of Americans needing vaccination to reach herd immunity ranges from 60 to 72% [13]. However, according to a November 2020 survey [14], 40% of Americans said that they will “definitely not” or “probably not” get the COVID-19 vaccine when it becomes available to them. Therefore, more needs to be done to bolster interest and trust in the vaccines. While companies and governmental organizations attempt to convey the necessary strategies to ease vaccine uncertainty and hesitation, a large segment of the lay public remains skeptical. As of May 2021, there were state-level COVID-19 vaccine incentives developed to increase vaccination rates across the United States. Irrespective of these incentives, only 48.6% of the US population was fully vaccinated as of July 2021, while 56% had received at least one dose [15]. Given these data, reasons surrounding vaccination hesitancy needed to be further explored. We aimed to expand current knowledge about COVID-19 vaccine perceptions through a characterization of sociocultural, socioeconomic, and demographic features in the context of opinions about receiving a COVID-19 vaccine. The objectives of the present survey were to establish:

1. What segments of the population believe the COVID-19 vaccines to be safe?
2. What are the perceived barriers to obtaining the COVID-19 vaccine—for self and others?
3. Is there an association between individual sociocultural characteristics and either acceptance or rejection of the vaccine?
4. Is there an association between individual demographic characteristics and either acceptance or rejection of the vaccine?

## Materials and methods

This research project was granted IRB approval by Indiana University (protocol #10670).

Data collection was done using an online survey distributed to the general public, and our methodology followed criteria from the CHERRIES checklist [16]. The survey was created using Qualtrics and piloted with 15 respondents. Based on responses and feedback from our iterative process to pilot the survey, questions were added, rephrased, or deleted. The final survey had 37 questions, with 1–6 questions per page. Question format included 28 multiple choices, with the remainder as yes/no questions. Both English and Spanish versions of the survey were available. A description of the ethical approval, anonymity, and data utilization was provided and acknowledged at the beginning of the survey. Personal information was not required, and participants were offered the option to enter an email address if they wished to participate in an optional raffle draw for five $20 Walmart gift cards. All data were stored in a secure password protected website, to which only study investigators had access. A completeness check prior to submission was not implemented, but a forced response feature on Qualtrics was used for all questions except those involving zip code and email address, to ensure that no significant questions were left unanswered. A link to the final version of the survey was posted to a Facebook page created for the study, and Facebook advertisements were used to promote the study. The survey was made available on March 19^th^, 2021 and was closed on April 30^th^, 2021. The final data collection survey is available as an attachment (S1 File).

This was a survey open to every Facebook user in the United States, based on the assumption that every adult was at risk of COVID-19 infection and should theoretically be able to get the vaccine. We limited responses to people stating they were at least 18-years old and able to read, understand, and agree to the terms of the online survey. Bivariate associations were evaluated using Mantel-Haenszel chi-square tests for questions where one or both variables had ordered categorical responses, and Pearson chi-square tests if both variables had nominal categories. Multivariable models were also performed, using an a priori p-value cut point of 0.20 for inclusion in the model. Collinearity between variables was assessed, leading to the exclusion of several variables from each multivariable model, retaining those based on statistical analysis and the team’s clinical experience. For ease of analysis, race was grouped into 2 categories: white and underrepresented minority. Low income was categorized based on respondents who indicated making less than $40,000 in annual income. The final level of significance for these multivariable models was set at p < 0.05.

All analytic assumptions were verified, and the analyses were performed using SAS/STAT software^®^ v9.4 [17].

## Results

A total of 2,626 responses were obtained. Based on a total of 3,743 potential participants who clicked our survey link on Facebook, our completion rate was 70.2%. Following data cleaning and exclusion of incomplete responses, a total of 2,259 responses were evaluable.

As outlined in Table 1, most participants were under 60 years of age (61.5%; median age in the 46–60 years group), female (79.2%) and white (89.6%). Most had never been employed in the healthcare field (63.4%), some were employed full time (44.5%), many had at least some college education (93.1%), about half were affiliated with the Democratic party (54.7%), and many lived within family households (75.7%).

**Table 1:** Descriptive statistics for variables in study.

**Demographics**	
Age (years)	
18–30	277 (12.3)
31–45	460 (20.4)
46–60	651 (28.8)
61–75	789 (34.9)
76–90	81 (3.6)
> = 91	1 (<0.1)
Healthcare Employee	
Current	348 (15.4)
Former	478 (21.2)
Never	1433 (63.4)
Gender	
Male	443 (19.6)
Female	1790 (79.2)
Other	7 (0.3)
Non-binary	19 (0.8)
Hispanic	127 (5.6)
Race	
White	2025 (89.6)
Black	33 (1.5)
Asian	88 (3.9)
American Indian/Alaskan Native	28 (1.2)
Native Hawaiian/Pacific Islander	10 (0.4)
Other	39 (1.7)
Multi	36 (1.6)
Race (2 categories)	
White	2025 (89.6)
Under-represented minority	234 (10.4)
Employed	
Yes, full time (> = 35 hours/week)	1005 (44.5)
Yes, part time	303 (13.4)
Yes, currently furloughed without pay	22 (1.0)
No, looking	55 (2.4)
No, not looking	682 (30.2)
Other	156 (6.9)
Yes, currently furloughed with pay	36 (1.6)
Income (%)	
0–9,999	157 (7.0)
10,000–19,999	175 (7.8)
20,000–29,999	193 (8.5)
30,000–39,999	191 (8.5)
40,000–49,999	208 (9.20
50,000–59,999	216 (9.6)
60,000–69,999	164 (7.3)
70,000–79,999	163 (7.2)
80,000–89,999	97 (4.3)
90,000–99,999	76 (3.4)
100,000+	313 (13.9)
Prefer not to answer	306 (13.6)
Median income by zip code	51,178 (1432–161,992); 42,771–66,971
Education	
Did not finish high school	12 (0.5)
High school diploma/GED	143 (6.3)
Some college or Associate’s degree	432 (19.1)
Bachelor’s degree	723 (32.0)
Graduate degree	949 (42.0)
Political Party	
Democrat	1236 (54.7)
Republican	407 (18.0)
Independent	510 (22.6)
Other	106 (4.7)
Living Situation	
Alone	418 (18.5)
Family	1710 (75.7)
Friends/roommates	131 (5.8)
**COVID questions**	
Received vaccination	1967 (87.1)
Which vaccination	
Pfizer	1045 (53.1)
Moderna	802 (40.8)
Unknown	11 (0.6)
Other	109 (5.5)
Select up to three reasons why you haven’t received the COVID-19 vaccination (these are all independent)	
Scheduled soon	61 (2.7)
No access	26 (1.2)
Not eligible	41 (1.8)
Do not think it is safe	93 (4.1)
Underlying conditions	73 (3.2)
Do not believe in vaccines	12 (0.5)
Cannot afford it	10 (0.4)
Other	33 (1.5)
Already infected so don’t need	10 (0.4)
Wait to see how it affects others first	104 (4.6)
Don’t have transportation	17 (0.8)
Do not believe I need the COVID vaccination	66 (2.9)
Belief in having had COVID	261 (11.2)
High risk conditions	1127 (49.9)
Think need for ICU	
Strongly agree	253 (11.2)
Somewhat agree	616 (27.3)
Somewhat disagree	901 (39.9)
Strongly disagree	489 (21.7)
Think will die	
Extremely Likely	95 (4.2)
Somewhat Likely	422 (18.8)
Somewhat Unlikely	907 (40.3)
Extremely Unlikely	826 (36.7)
Think will transmit	
Extremely Likely	1114 (49.3)
Somewhat Likely	845 (37.4)
Somewhat Unlikely	226 (10.0)
Extremely Unlikely	74 (3.3)
Vaccines safe	
Strongly agree	1570 (69.5)
Somewhat agree	521 (23.1)
Somewhat disagree	115 (5.1)
Strongly disagree	53 (2.4)
Trust science	
Strongly agree	1655 (73.3)
Somewhat agree	444 (19.7)
Somewhat disagree	105 (4.7)
Strongly disagree	55 (2.4)
Trust doctors	
Strongly agree	1628 (72.1)
Somewhat agree	462 (20.5)
Somewhat disagree	114 (5.1)
Strongly disagree	55 (2.4)
Flu shot	1783 (78.9)
Flu shot—why not (independent options)	
Do not believe in vaccines in general	32 (1.4)
Do not believe I need flu shot	245 910.9)
Do not think flu shot is safe	42 (1.9)
Underlying health conditions	66 (2.9)
Cannot afford	34 (1.5)
No access	27 (1.2)
No transportation	10 (0.4)
other	134 (5.9)
Will obtain COVID shot yearly if CDC recommends	
Extremely likely	1723 (76.3)
Somewhat likely	307 (13.6)
Somewhat unlikely	108 (4.8)
Extremely likely	121 (5.4)
Shot administrator NOT comfortable with (independent options)	
Doctors	126 (5.6)
Nurses	119 (5.3)
Pharmacists	229 (10.1)
Medical students	338 (15.0)
Dentists	796 (35.2)
Dental hygienists	1181 (52.3)
Non-healthcare workers	1862 (82.4)
Other	248 (11.0)
Face covering effectiveness	
Extremely effective	1174 (52.0)
Somewhat effective	896 (39.7)
Somewhat ineffective	101 (4.5)
Extremely ineffective	88 (3.9)
Social Distancing effectiveness	
Extremely effective	1310 (58.0)
Somewhat effective	816 (36.1)
Somewhat ineffective	92 (4.1)
Extremely ineffective	40 (1.8)
Hand Washing effectiveness	
Extremely effective	1266 (56.0)
Somewhat effective	853 (37.8)
Somewhat ineffective	116 (5.1)
Extremely ineffective	24 (1.1)
Return for a second dose	
Extremely likely	444 (19.7)
Somewhat likely	68 (3.0)
Somewhat unlikely	13 (0.6)
Extremely unlikely	9 (0.4)
Do not plan to get vaccine	150 (6.6)
Already received second dose	1482 (65.6)
Received single dose vaccine	93 (4.1)
I don’t have to get vaccine if others do	
Strongly agree	55 (2.4)
Somewhat agree	203 (9.0)
Somewhat disagree	415 (18.4)
Strongly disagree	1586 (70.2)
I want to protect others	
Strongly agree	1824 (80.7)
Somewhat agree	256 (11.3)
Somewhat disagree	101 (4.5)
Strongly disagree	78 (3.5)
Do you know anyone who got COVID vaccine	2201 (97.4)
Vaccinated if friends/family do	
Extremely likely	89 (30.5)
Somewhat likely	53 (18.2)
Somewhat unlikely	60 (20.6)
Extremely unlikely	90 (30.8)
Reliable sources for vaccination	
Doctors/nurses/other healthcare	2067 (91.5)
Family members	488 (21.6)
Friends	150 (6.6)
Religious community group	31 (1.4)
Peers from same racial/ethnic group	32 (1.4)
Magazines/newspaper/radio	57 (2.5)
News websites	257 (11.4)
Social media websites	47 (2.1)
Health information websites	1269 (56.2)
Government sources	759 (33.6)
Peer reviewed journals	949 (42.0)
Celebrities	10 (0.4)

Values are frequencies (percentages). Frequencies may not add to sample total due to missing response data. Values for median income (range); IQR.

To determine what groups perceived the vaccine as safe, bivariate and multivariable models were created. Table 2 shows that subjects who perceived the vaccination as being safe were more likely to have already obtained their second dose or planned on getting it (we allowed for single shot vaccines in our analyses) (97% vs. 12%; p< 0.0001), did not have a prior health condition (98% vs. 86%; p< 0.0001), trusted science (97.1% vs. 21%; p< 0.0001)/vaccines (97% vs. 17%; p< 0.0001)/doctors (97% vs. 21%; p< 0.0001), believed in the effectiveness of hand washing (94% vs. 88%; p = 0.0056)/social distancing (96% vs. 59%; p< 0.0001)/wearing a mask (95% vs. 43%; p< 0.0001), were female (88% vs. 66%; p = 0.0005), were white (90% vs. 82%; p = 0.0063), had higher levels of education (94% vs. 79%; p< 0.0001), and identified as Democrats (58% vs. 7%; p< 0.0001). In the multivariate model, subjects who were still independently associated with the perception of the vaccines being safe were those more likely to have received their second dose (or planned on it) (p< 0.0001), who trusted science (p< 0.0001), had higher levels of education (p = 0.0007), or were Democrats (p = 0.0020).

**Table 2:** Bivariate analysis for variables associated with perceived vaccine safety.

	Bivariate odds ratios	Multivariable odds ratios
Second dose		
Have 2^nd^ dose	245.06 (135.08, 444.59); p < .0001	40.03 (19.03, 84.21); p < .0001
Unlikely	reference	Reference
Trust Science		
Agree	129.40 (78.38, 213.64); p < .0001	10.54 (5.10, 21.77); p < .0001
Disagree	Reference	Reference
Healthcare Field		
Currently employed	Reference	Reference
Not currently employed	0.71 (0.35, 1.44); p = .9017	1.52 (0.50, 4.61); p = .1633
Never	0.53 (0.29, 0.99); p = .0314	0.73 (0.27, 1.97); p = .1353
Gender		
Female	Reference	Reference
Male	0.47 (0.32, 0.70); p = .4980	0.43 (0.21, 0.88); p = .1962
Other	0.12 (0.02, 0.63); p = .0385	1.13 (0.10, 12.53); p = .6608
Race		
White	1.94 (1.20, 3.14); p = .0072	1.46 (0.64, 3.31); p = .3648
Under-represented minority	Reference	Reference
Education		
< = high school	0.09 (0.05, 0.17); p < .0001	0.15 (0.05, 0.44); p = .0007
Some college/AS	0.15 (0.08, 0.26); p < .0001	0.42 (0.17, 1.03); p = .7048
BS/BA	0.37 (0.21, 0.67); p = .0356	0.72 (0.29, 1.79); p = .1022
Graduate degree	Reference	Reference
Political party		
Democrat	Reference	Reference
Republican	0.05 (0.02, 0.09); p < .0001	0.18 (0.07, 0.47); p = .0020
Other	0.07 (0.03, 0.14); p < .0001	0.28 (0.11, 0.73); p = .2523

Values are odds ratio (95% confidence intervals) with p-value from logistic regression models for being in the group “vaccines are safe.” Bivariate are given to be a direct comparison with the multivariable odds ratios. Variables were chosen based on bivariate association strength (p<0.20), but then pared down due to high collinearity among those variables.

To determine what groups were likely to perceive the most barriers to vaccination, bivariate and multivariable models were created (Table 3). By analyzing subjects who were actively seeking vaccination versus those who were not, we found the former were more likely to have had their second dose (or were likely to get it) (92% vs. 20%; p< 0.0001), did not have a prior health condition (94% vs. 85%; p = 0.0283), trusted science (96% vs. 34%; p< 0.0001)/vaccines (95% vs. 31%; p< 0.0001)/doctors (93% vs. 35%; p< 0.0001), believed in the effectiveness of social distancing (91% vs. 68%; p< 0.0001)/wearing a mask (97% vs. 52%; p< 0.0001), were younger (p< 0.0001), were not male (72% vs. 68%; p = 0.0326), were an under-represented minority (40% vs. 23%; p = 0.0043), had a higher median income ($56,000 vs. $49,000; p = 0.0053), or were Democrats (48% vs. 12%; p< 0.0001). In the multivariate model, subjects that were still independently associated with actively seeking a vaccination were those with their second dose already received (or planned on it) (p< 0.0001) and who trusted in science (p = 0.0006).

**Table 3:** Bivariate analysis for variables associated with perceived barriers to vaccination.

	Bivariate odds ratios	Multivariable odds ratios
Second dose		
Have 2^nd^ dose	48.65 (20.78, 113.92); p < .0001	23.06 (7.38, 72.04); p < .0001
Unlikely	reference	Reference
Underlying Health Condition		
Yes	0.33 (0.12, 0.89); p = .0278	0.33 (0.08, 1.33); p = .1173
No	reference	
Trust Science		
Agree	42.81 (15.02, 121.98); p < .0001	12.33 (2.96, 51.40); p = .0006
Disagree	Reference	Reference
Age		
18–30	6.80 (3.11, 14.89); p < .0001	0.42 (0.09, 1.87); p = .4937
31–45	2.21 (0.97, 5.03); p = .6570	0.17 (0.03, 0.80); p = .0745
46–60	0.93 (0.36, 2.37); p = .0099	0.16 (0.03, 0.84)); p = .1193
61+	reference	reference
Gender		
Female	Reference	Reference
Male	0.89 (0.51, 1.56); p = .0305	0.52 (0.21, 1.31); p = .0553
Other	6.30 (1.24, 32.14); p = .0220	11.48 (0.38, 351.64); p = .1147
Race		
White	0.44 (0.26, 0.76); p = .0072	0.58 (0.22, 1.51)); p = .2640
Under-rep minority	Reference	Reference
Education		
< = high school	0.58 (0.24, 1.40); p = .1832	0.53 (0.13, 2.13); p = .1460
Some college/AS	0.69 (0.34, 1.43); p = .3621	0.96 (0.29, 3.26); p = .9865
BS/BA	1.23 (0.60, 2.55); p = .0646	1.74 (0.50, 6.08); p = .1193
Graduate degree	Reference	Reference
Political party		
Democrat	Reference	Reference
Republican	0.16 (0.08, 0.31); p = .0025	0.45 (0.14, 1.43); p = .6000
Other	0.15 (0.07, 0.28); p = .0005	0.33 (0.11, 0.96); p = .0985
Living Situation		
Lives Alone	0.34 (0.12, 0.95); p = .1115	0.50 (0.09, 2.66); p = .2449
Lives with family	0.43 (0.19, 0.96); p = .2936	1.19 (0.31, 4.60); p = .3125
Lives with friends/roommates	Reference	Reference

Values are odds ratios (95% confidence intervals) with p-values from logistic regression models, for being in the group “actively seeking vaccine.” Bivariate are given to be a direct comparison with the multivariable odds ratios. Variables were chosen based on bivariate association strength (p < .20), but then pared down due to high collinearity among those variables. In the event of such collinearity, I also kept the variables used in previous models (e.g. education rather than income).

Data for the final two objectives were aggregated and analyzed together (Table 4). For those who “do not believe in vaccines”, the variables more likely associated with such outcome included not having a high-risk medical condition (42% vs. 53%; p = 0.0111), not knowing someone who is vaccinated (87% vs. 98%; p< 0.0001), not trusting vaccines (21% vs. 97%; p< 0.0001)/science (26% vs. 97%; p< 0.0001)/doctors (28% vs. 97%; p< 0.0001), not believing in the effectiveness of hand washing (90% vs. 94%; p = 0.0410)/ social distancing (65% vs. 96%; p< 0.0001)/wearing a mask (51% vs. 94%; p< 0.0001), not receiving an annual flu shot (21% vs. 83%; p< 0.0001), thinking there is no need if others have been vaccinated (58% vs. 8%; p< 0.0001), and not wanting to get vaccinated to help others (27% vs. 96%; p< 0.0001). In the multivariate model, subjects that were still independently associated with not believing in vaccines did not know someone who was vaccinated (p< 0.0001), did not trust science (p< 0.0001), believed vaccination is unnecessary if others were vaccinated (p = 0.0007), and would not get vaccinated to help others (p< 0.0001).

**Table 4:** Association of population characteristics with either acceptance or rejection of vaccines.

	Bivariate odds ratios	Multivariable odds ratios
High Risk Condition		
Yes	0.62 (0.43, 0.90); p = .0117	0.91 (0.52, 1.59); p = .7346
No	reference	Reference
Know someone who is vaccinated		
Yes	0.13 (0.07, 0.23); p < .0001	0.14 (0.06, 0.36); p < .0001
No	Reference	Reference
Trust Science		
Agree	0.01 (0.01, 0.02); p < .0001	0.03 (0.02, 0.07); p < .0001
Disagree	Reference	Reference
No need to vaccinate if others have		
Agree	15.05 (10.36, 21.87); p < .0001	2.80 (1.54, 5.09); p = .0007
Disagree	Reference	Reference
Vaccinated to help others		
Agree	0.02 (0.01, 0.02); p < .0001	0.13 (0.07, 0.24); p < .0001
Disagree	Reference	Reference
Would if friends/family have been vaccinated		
Likely	0.16 (0.10, 0.27); p < .0001	0.09 (0.04, 0.20); p < .0001
Unlikely	Reference	Reference
Gender		
Male	1.86 (1.27, 2.73); p = .3111	0.79 (0.36, 1.73); p = .8410
Female	Reference	Reference
Other	1.52 (0.35, 6.53); p = .8838	0.83 (0.07, 9.39); p = .9524
Race (2 categories)		
White	0.31 (0.21, 0.47); p < .0001	0.50 (0.18, 1.33); p = .1644
Under-represented minority	Reference	Reference
Employed		
Full Time	0.67 (0.46, 0.96); p = .0270	0.71 (0.34, 1.51); p = .3763
Other	Reference	Reference
Median income by zip code (per $10,000)	0.80 (0.70, 0.92); p = .0013	0.77 (0.63, 0.94); p = .0106
Political Party		
Democrat	Reference	Reference
Republican	13.52 (7.57, 24.15); p < .0001	2.94 (1.03, 8.42); p = .0844
Other	9.27 (5.23, 16.42); p < .0001	2.18 (0.82, 5.83); p = .5115

Values are odds ratios (95% confidence intervals) with p-values from logistic regression models, for being in the group “do NOT believe in vaccines.” Bivariate are given to be a direct comparison with the multivariable odds ratios. Variables were chosen based on bivariate association strength (p < .20), but then pared down due to high collinearity among those variables.

Additionally, the variables associated with subjects who “do not believe in vaccines” included not getting vaccinated even if friends and family had been vaccinated (26% vs. 89%; p< 0.0001), being male (30% vs. 19%; p = 0.0053), being an underrepresented minority (25% vs. 9%; p< 0.0001), not being employed full time (65% vs. 55%; p = 0.0260), having a lower median income ($ 49, 000 vs. $51, 000; p = 0.0020), having lower levels of educational attainment (21% vs. 6%; p< 0.0001), and not being a Democrat (89% vs. 43%; p< 0.0001). In the multivariate model, subjects who were still independently associated with not believing in vaccines were those not getting vaccinated even if friends and family had done so (p< 0.0001), and having a lower median income (p = 0.0106).

## Discussion

Our study is not the first to examine the relationship between various demographics and vaccine hesitancy. Kini and colleagues explored 39 studies regarding demographics of vaccine acceptance and hesitation. Their systematic review suggests that vaccine acceptance increases with age and is higher for males and white individuals [18]. While our study reports different significant findings (see below), this is likely attributed to the context and sample of the studies, along with possible confounding variables as discussed later. Our results pertain to the time when data were collected: given the long and haphazard evolution of the pandemic and associated perceptions, the relevance of our results must be contextualized to the time and the stage of the pandemic. Our data show some disparities in perception and opinions regarding the COVID-19 vaccines based on the following key variables: age, race, income, educational level, underlying health conditions, and political partisanship. Participants who had received the first of two doses of the COVID-19 vaccine at the time of our study may already have been convinced of the safety of the vaccines. Additionally, during the early stages of vaccine promotion, there was emphasis from the CDC on possible worsening of underlying pulmonary, cardiac, and other health conditions, such as chronic obstructive pulmonary disease, heart failure, and asthma [19]. This could explain why individuals with underlying health conditions were likely to regard the vaccines as protective and safe.

Our results also showed that those who identifies as white, compared to members of underrepresented minorities, were more likely to consider the vaccine as safe. Based on an assumption of a positive correlation between perceiving the vaccine as safe and actually getting the vaccine, the CDC has shown that as of July 4^th^, 2021, of those who had received at least one dose of the vaccine, 59% were white, 9% were black, 16% were Hispanic/Latinx, and 6% were Asian Americans [20]. However, it is unclear whether such disparity is affected by the communities in which vaccines are most readily available, or if such disparity in fact represents an individual decision due to distrust that might exist between underrepresented minorities and the healthcare industry. As such, it is vital to review past literature as it pertains to recent findings during the pandemic. Regarding vaccine hesitancy of underrepresented minorities, there has been clear evidence of disparities in healthcare treatment for Black and white patients. Davidio et al reviewed multiple papers that describe physician perceptions and treatment of Black vs. white patients with clear significance regarding the negative handling of Black patients [21]. Armstrong et al point out that experience of discrimination was strongly associated with healthcare system distrust (HCSD) in their study comparing African American and white survey respondents [22]. Additionally, Balasuriya et al explored factors associated with COVID-19 acceptance and access among Black and Latinx communities, and identified the pervasive mistreatment of Black and Latinx communities, rooted in structural racism, to be a key influence on vaccine acceptance [23]. Results such as this provide a strong basis to argue why underrepresented minorities may have been less eager to seek out vaccinations. Regarding vaccine hesitancy and political affiliation, other studies corroborate these results. In one study, it was found that US Republican counties consistently had lower general vaccination rates than Democratic counties [24]. In a polling done by Kaiser Family Foundation in May 2020, it was found that Republicans were less likely to report wearing masks, social distancing or getting vaccinated against COVID-19 [25].

Level of education has a strong effect on willingness to receive a COVID-19 vaccine: having a college degree has been associated with a 43% increase in likelihood of getting the vaccine [26]. Assuming the likelihood of obtaining the COVID-19 vaccine is positively correlated with perception that the vaccine is safe, it is worthwhile posing the question whether level of education outweighs other effects of race, gender, political affiliation, and underlying health conditions. Delay in COVID-19 vaccination notwithstanding (earlier in 2021 when our data were collected), the CDC has pointed to a divide in communities based on political party affiliation. To ultimately determine the prime factors in safety perception, we conducted a multivariable analysis and found that the following groups were most likely to perceive vaccines as being safe: 99.3% Democrats (vs. 86.0% Republicans, specifically) and 93.1% with higher educational attainment (vs. 6.8% with high school level specifically). It is important to correlate these results with previous studies that examined similar topics. Regarding results about education impacting vaccine rates, previous studies would support this. Suryadevara and colleagues collaborated with their county health department to educate high-risk, resource-poor families regarding vaccination concerns. Their results showed a drastic increase for general vaccine completion and annual influenza vaccine rates [27]. Another study showed that when providing low-literacy educational materials to resource-poor families regarding the pneumococcal vaccine, the test group was four times more likely to discuss the vaccination in appointments and five times more likely to receive the vaccine than control group [28]. Even more recent studies with COVID-19 support our findings. For instance, a recent study indicated that lack of high school education positively correlates with increased vaccine hesitancy and decreased vaccination levels [29].

Our multivariable model outcome also suggests that race and ethnicity are not necessarily the primary determinants of vaccine hesitancy and likelihood of vaccination, because low vaccination rates among underrepresented populations may be explained by the historical distrust within some members of underrepresented minorities toward health care organizations and providers, as well as suspicion about clinical research studies, in view of past atrocities such as the Tuskegee Syphilis experiment [30], or similar experiments with STD infections in Guatemala [31]. Our multivariable results support this possibility by indicating those being potential factors in rejecting the COVID-19 vaccine. Specifically, after adjusting for variables, one of the groups found to be independently associated and most likely to reject vaccination according to socioeconomic and demographic factors were individuals with lower income. Considering that low-income populations usually consist of groups that identify as underrepresented minorities [32], slow rates of vaccination in these groups might reflect individual distrust of health care providers. However, this finding does not rule out the possibility of low distributions in low-income locations (e.g., rural), which could be a barrier by itself for vaccination opportunities. As pointed out by DeMaria-Ghalili and colleagues, “health inequalities are most acute among those living in rural and low resourced areas of the state, and among underrepresented populations (particularly Black/African American and Latino), who lack access to health care, experience digital divide, and face persistent local healthcare workforce shortages.” The report further discusses that people in areas of lower socio-economic status or fewer resources (usually rural areas) have a more difficult time scheduling and going to appointments for vaccinations, noting “pharmacy deserts” to be an issue in having access to appropriate healthcare resources such as vaccines [33]. Economic precarity and poor technological advancements may be obstacles to both registering for and getting the vaccine, possibly associated with sparse information among low-income populations [34]. Therefore, to bolster vaccination, efforts should be made to target groups who are most likely to encounter barriers to COVID-19 vaccination, through governmental incentives, including free childcare and rides to vaccination sites, lottery tickets or cash vouchers, complimentary food and drinks at the vaccination sites, and tax credit [35], rather than privately offered incentives that may vary greatly throughout the country.

Our successful recruitment for this survey was helped by the ever-increasing prevalence of social media in peoples’ lives. This highlights the need for proper, scientific-based information regarding the pandemic to reach the lay public before opinions appear on social media newsfeeds. On the other hand, only 2.1% of our sample thought that social media sites were reliable sources for vaccine information. While this would appear to suggest limited influence of social media with regard to COVID vaccines, we have to interpret this with caution in view of a small, self-selected sample that may not reflect the U.S. population as a whole. While some individuals may have legitimate reasons for declining vaccination, e.g. allergies to some ingredients in the preparation or other medical contraindications, misperceptions about vaccines as presented by some members of the media can lead to vaccine refusal for inappropriate reasons [36]. Therefore, it is important to disseminate the scientific basis for vaccines whenever possible. Negative press about variant viruses and the possibility of ineffective vaccines lead to further public distrust of the otherwise monumental feat of creating and distributing the COVID-19 vaccines [37]. Education of the public is essential for the continued success of vaccination efforts in general. As an example, in one study [38], Human Papilloma Virus (HPV) vaccine education sessions were held for parents, healthcare and school staff who had little knowledge regarding HPV vaccines. After the sessions, results showed that over 90% of respondents felt vaccine education was important and 85–97% were supportive of school-based vaccine clinics. In another study on flu vaccination during pregnancy [39], pregnant women refused flu vaccines due to likely susceptibility to influenza and concerns for vaccine safety. The study intervention was a brief educational video by the CDC, which addressed vaccination health beliefs in a clear and easy to understand format. The primary outcome was receipt of the flu vaccine on the next prenatal visit, and suggested that appropriate education and reassurance were influential in vaccination. We must do the same for the COVID-19 vaccine, seeing that our findings suggest that educational attainment is one of the two most important factors that determine the likelihood that one will perceive the vaccine as safe and be likely to accept vaccination. Given that an overwhelming majority of our respondents indicated that they considered doctors, nurses, and other healthcare workers as reliable sources of vaccination information, it is imperative to begin incorporating COVID-19 vaccine questions and education during health care visits. Moreover, training healthcare professionals in cultural competency, defined as “the ability of individuals and systems to work or respond effectively across cultures in a way that acknowledges and respects the culture of the person or organization being served” [40] would help them navigate this conversation with knowledge and transparency to promote mutual trust and possibly increased likelihood of vaccination [41, 42]. Unfortunately, cultural competency training is still limited in medical schools and residency programs [43], and broader implementation is needed. This will be critical for engaging minority/underrepresented groups, though we acknowledge that these groups may have general difficulties accessing any medical care and this in turn may contribute to lower vaccination rates. Some respondents chose “no access” as a reason for not receiving the vaccine. The term “no access” is admittedly broad and could have included decreased vaccination distribution to impoverished neighborhoods, or it could mean that individuals do not know where to go to get their vaccine. We kept our questionnaire concise so as not to overburden respondents, and consequently could not necessarily qualify the specific reasons for perception about no or limited access. Further investigation is needed to characterize the specific obstacles experienced by people seeking the vaccination. As health literacy regarding the still relatively new COVID-19 pandemic remains a challenge [44], our present survey can hopefully act as a compass to inform providers on the underlying rationale that their patients have for being skeptical about vaccines or medical advice.

In addition, we need steps to encourage the population to get vaccinated irrespective of political affiliation. Per our findings, those who identify as Democrats are more likely to perceive the vaccine as being safe. Partisanship and vaccination status continue to play a role in both U.S. vaccination efforts and the government’s response to the pandemic in general. Other studies have shown similar results [45], where 65% of Democrats and 51% of vaccinated adults say that the surge in COVID cases makes them angry at people who have not gotten a vaccine, while 59% of Republicans and 56% of unvaccinated adults say that the federal government should be blamed. Our study shows that Republicans less likely to become vaccinated trust information that comes directly from their health care team, more than information that originates from the government. Therefore, ensuring that all personnel on the health care team are culturally competent to facilitate conversations brought on by patients regarding the COVID-19 vaccine will be instrumental in ensuring vaccination acceptance across spectra. Finally, incentives must be focused on core groups that we believe are more likely to reject the vaccine. These include underrepresented minorities, people with lower educational level, those who identify as young, males, and those with high risk underlying medical conditions.

Our study has limitations, especially regarding data collection. Given the current pandemic and difficulty with in-person survey distribution, it was decided that an online distribution would be preferable, based on the assumption that every individual is at risk of contracting the virus and becoming affected by the pandemic. We used Facebook due to its wide reach. However, we recognize that not everyone has access to computers or Facebook, so this survey may favor those of higher socioeconomic status. Likewise, we did not seek parity since the sample was largely one of convenience, based on who responded to the questionnaire. Although forced responses were used for our survey to ensure completion and prevent answers, we could not determine other potential factors that may have caused incomplete responses in cases where respondents were allowed to select up to three options, e.g. for trusted sources of information. Obstacles to completion might have included feeling pressed for time, concerns about privacy in view of the open nature of social media, or rejection based on personally held political views. This could result in a self-selection bias due to differences between respondents and non-respondents, therefore skewing the findings. For example, many participants were white, female, and/or Democrat voters, which is not representative of the U.S. population per se and could bias the results in favor of opting for vaccination, perhaps due to stronger belief in vaccines. Obviously, given the enormous number of Facebook users in the U.S., and the fact that users are allowed to protect their privacy by restricting access to personal data (including by omitting it in their profiles), it would be difficult to assess the “typical” Facebook user in the context of these factors. Along those lines, about 87% of respondents were already vaccinated, which suggests that most considered the benefits greater than the risks. This may therefore result in under-reporting and under-characterizing negative views of the vaccine that we sought to capture in the survey. Another limitation of this study is that it only represents a snapshot in time of opinions of COVID-19 vaccine perceptions, which can be fluid. Because the vaccine data are rapidly changing and information provided to the public may evolve as days progress, our results can only be applicable to this specific point in time. Ideally, the present study should be repeated in the future to ascertain trends over time. From a methodological standpoint, future studies should focus on obtaining a wider and more diverse set of respondents, including individuals that do not have access to computers or Facebook. One feasible alternative could be the distribution of both online and paper surveys to the same group of respondents during the same wave of data collection, thus allowing for estimation of changes across strategies for survey contact.

While our findings are in line with some existing perspectives in the field, as they relate to the role of socioeconomic factors [26, 32], educational influence [38, 39], and partisanship [45], we have contributed a more robust and elaborate perception of the U.S population on COVID vaccines, while identifying specific groups at risk for not getting a vaccine. In conclusion, level of education and partisanship, but not race/ethnicity, were the most likely factors associated with vaccine hesitancy or likelihood to vaccinate. This suggests that improved education, not just about vaccines per se, but with regard to formal schooling in general, may be at the heart of promoting greater acceptability of vaccination. Likewise, low vaccination rates among underrepresented minorities may be due to distrust for healthcare industries, but further research is needed to fully characterize the relative contributions of low access vs. distrust. Many white people and many with a Republican party affiliation also expressed reluctance about vaccination, suggesting that mistrust of the healthcare industry, vaccinations in general, and/or the government is not limited to minorities and/or economically challenged populations. Regardless, population sub-groups less likely to be vaccinated and/or receptive to vaccines should be targeted for vaccine education and incentives, and outcomes of these interventions need to be closely studied for determination of efficacy.

## Supporting information

S1 File Qualtrics survey questionnaire. (PDF)

S1 Data Inclusion criteria. (DOCX)
