# Supplementary material for: COVID-19 vaccine: A 2021 analysis of perceptions on vaccine safety and promise in a U.S. sample
Source: PLoS One. 2022 May 19;17(5):e0268784. doi: 10.1371/journal.pone.0268784 (PMC9119541; doi:10.1371/journal.pone.0268784)
Supplement: S1 File — (PDF) [file pone.0268784.s001.pdf]

Informed Consent for Participation

COVID-19 Vaccines: Analysis of Perceptions on Vaccine Safety and Promise

What is the purpose of this study, and what will it involve?

This study on COVID-19 vaccine perceptions is a research survey about your experiences and thoughts regarding the current COVID-19 vaccines. You do not need any special knowledge of COVID-19 or the vaccines to complete this survey. This study is primarily being conducted by researchers at Indiana University Fairbanks School of Public Health (IUFSPH). We are inviting you to take the survey because you are at least 18 years of age and live in the United States. This survey will take about 10 minutes to complete. Our research is designed to gain new knowledge on both COVID-19 and the newly FDA approved COVID-19 vaccines. All information collected will be kept confidential and will only be accessible to members of the research team. We do not anticipate any major risk to you by participating in this study; one possible minor risk may include discomfort about answering some questions. With some studies, there is concern of loss of confidentiality. However, your data will be stored on a secure, password protected website to which only the investigators will have access. No information which could identify you will be shared in publications about this study; no Private Health Information (PHI), such as name or address, is required on this survey, and all answers are anonymous. The information you provide in this survey will be very valuable to our research and our understanding of population behaviors and attitudes towards the COVID-19 vaccines. If you would like a copy of the final results of the study, we will be happy to send them to you.

Is there any compensation for participation in this study?

Of the approximately 3000 participants who completes this survey in its entirety, five people will be eligible to win a $20 Walmart gift cards via a raffle draw. Therefore, if you would like to participate, please add your email address at the end of the survey in order for us to contact you if you win the raffle.

Your participation in this study is voluntary.

You may choose not to take part in this survey study. However, if you do decide to participate, you may still decide to withdraw at any point. If you do choose to withdraw from the survey early, you will unfortunately be unable to enter your email for the raffle. At the end of the survey you will be asked to submit your results, at which point it will not be possible to withdraw your data. If you have any questions or concerns about this study, you may contact the researchers at vaxc19a@iu.edu. For questions about your rights as a research participant or to discuss any problems, complaints or concerns about a research study, or to obtain information, or offer input, contact the Indiana University Human Subjects Office at (317) 278-3458 or (800) 696-2949.

Once you have read above, please select "I agree" below to continue with the survey.

- ○ I agree

Study Requirements

How old are you? (select one)

- ○ Younger than 18

- ○ 18-30
- ○ 31-45
- ○ 46-60
- ○ 61-75
- ○ 76-90
- ○ 91 or older

In order to participate in this survey, you must be 18 years of age or older. Because you selected "Younger than 18" in the previous question, you do not qualify to participate in the remainder of this survey.

COVID-19 Vaccination Status

Have you received a COVID-19 vaccine? (Select one)

- ○ Yes
- ○ No

Which vaccine did you receive? (Select one)

- ○ Pfizer

- ○ Moderna
- ○ Unknown
- ○ ☐ Other (please specify)

From the options below, please select up to 3 reasons why you have not yet received the COVID-19 vaccine?

- ☐ I am scheduled to get the vaccine soon.
- ☐ I am eligible for the vaccine but do not have access to it.
- ☐ I am eligible for the vaccine but do not have transportation to get it.
- ☐ I am eligible for the vaccine but cannot financially afford it.
- ☐ I am not eligible to get the vaccine yet.
- ☐ I do not believe I need the COVID vaccine in general.
- ☐ I have already been infected with COVID-19 and therefore don't believe I need the vaccine.
- ☐ I do not believe in vaccines.
- ☐ I do not think the COVID vaccine is safe.
- ☐ I am nervous that the vaccine would affect my underlying allergies or health conditions.
- ☐ I would like to wait and see how the vaccine affects others first.
- ☐ Other (please specify)

Threat of Infection

Have you been officially diagnosed with COVID-19, either recently or in the past? (Select one)

- Yes
- No

Even without official diagnosis, do you believe that you may have gotten COVID-19 based on symptoms? (Select one)

- Yes
- No
- Unsure

Do you have any health condition that could make infection with COVID-19 more severe (e.g. medical history of immunosuppression, asthma, COPD, heart disease, diabetes, obesity, etc.)?

- Yes
- No
- Unsure

Select how much you agree or disagree with the following: "I think that I will be admitted to the intensive care unit (ICU) if I get infected with COVID-19" (Select one)

- Strongly agree
- Somewhat agree
- Somewhat disagree
- Strongly disagree

Select how much you agree or disagree with the following: "I think that I will die if I get infected with COVID-19" (Select one)

- Strongly agree
- Somewhat agree
- Somewhat disagree
- Strongly disagree

If you were to test positive for COVID-19 and interact with family or friends, how likely do you think you would be to transmit the virus to them? (Select one)

- Extremely likely
- Somewhat likely
- Somewhat unlikely

- Extremely unlikely

COVID Vaccine Perception

How much do you agree with the following statement: "The COVID-19 vaccines are safe." (Select one)

- Strongly agree
- Somewhat agree
- Somewhat disagree
- Strongly disagree

How much do you trust the science and research that tells us that the vaccines are effective against COVID-19? (Select one)

- Strongly trust
- Somewhat trust
- Somewhat distrust
- Strongly distrust

How much do you trust the doctors who tell us that the vaccines are effective against COVID-19? (Select one)

- ○ Strongly trust
- ○ Somewhat trust
- ○ Somewhat distrust
- ○ Strongly distrust

Do you usually receive an annual or yearly flu vaccine? (Select one)

- ○ Yes
- ○ No

If no to the previous question, why not? (Please select up to 3 responses)

- ☐ I do not believe in vaccines in general.
- ☐ I do not believe I need the flu vaccine.
- ☐ I do not think the flu vaccine is safe.
- ☐ I do not think the vaccine would be safe for me, due to underlying allergies and health risks.
- ☐ I cannot financially afford the vaccine.
- ☐ I do not have access to the vaccine.
- ☐ I do not have transportation to get the vaccine.
- ☐ ______ Other (please specify)

How likely are you to get a COVID vaccine every year if the CDC were to recommend it? (Select one)

- Extremely likely
- Somewhat likely
- Somewhat unlikely
- Extremely unlikely

From which of the following people, if any, would you NOT feel comfortable receiving a COVID vaccine? (Select all that apply)

- Doctors
- Nurses
- Pharmacists
- Medical students
- Dentists
- Dental Hygenists
- Non-healthcare workers
- Other

How effective do you believe that face coverings are in protecting against COVID-19? (Select one)

- Extremely effective
- Somewhat effective
- Somewhat ineffective
- Extremely ineffective

How effective do you believe that social distancing is in protecting against COVID-19? (Select one)

- Extremely effective
- Somewhat effective
- Somewhat ineffective
- Extremely ineffective

How effective do you believe that hand-washing is in protecting against COVID-19? (Select one)

- Extremely effective
- Somewhat effective
- Somewhat ineffective
- Extremely ineffective

The currently available COVID-19 vaccines require two vaccinations that are either 3 or 4 weeks apart. How likely

are you to return for the second dose? (Select one)

- I have already received my second dose
- Extremely likely
- Somewhat likely
- Somewhat unlikely
- Extremely unlikely
- Not applicable; I do not plan to get the vaccine at this time

How much do you agree with the following statement: "In general, when most other people are vaccinated, I don’t have to get vaccinated." (Select one)

- Strongly agree
- Somewhat agree
- Somewhat disagree
- Strongly disagree

How much do you agree with the following statement: "I would get vaccinated so that I can help protect people with a weaker immune system." (Select one)

- Strongly agree
- Somewhat agree
- Somewhat disagree

- ○ Strongly disagree

External Influences Towards Vaccines

Do you personally know anyone who has received the COVID-19 vaccine? (Select one)

- ○ Yes
- ○ No

How likely would you be to get vaccinated if your family or friends got the vaccine? (Select one)

- ○ Extremely Likely
- ○ Somewhat likely
- ○ Somewhat unlikely
- ○ Extremely unlikely

From the list below, please select up to 3 of the most reliable sources for whether or not you should receive the COVID-19 vaccine.

- ☐ Doctors/Nurses/Other healthcare workers

- ☐ Family (including spouse, significant other, domestic partner, guardian or parent, extended family member, etc)
- ☐ Friends
- ☐ Religious community group
- ☐ Peers from my same racial/ethnic group
- ☐ Magazines, newspapers, and radio stations
- ☐ News websites
- ☐ Social media websites
- ☐ Health information websites
- ☐ Government sources
- ☐ Peer reviewed journal articles
- ☐ Celebrity/Public figure

Additional Demographic Information

Are you currently or have you ever been employed in the healthcare field? (For example, are you a physician, nurse, healthcare administrator or clinical support staff?)

- ○ Currently employed in the healthcare field
- ○ Not currently employed in the healthcare field but have been in the past
- ○ Have never been employed in the healthcare field.

What is your gender? (Select one)

- ○ Male
- ○ Female
- ○ Non-binary
- ○ [ ] Other

Are you Hispanic or Latino/a? (Select one)

- ○ Yes
- ○ No

Which of the following best describes your race? (Select all that apply)

- ☐ American Indian or Alaskan Native
- ☐ Native Hawaiian or Pacific Islander
- ☐ Asian
- ☐ White or Caucasian
- ☐ Black or African American
- ☐ [ ] Other

Are you currently employed? (Select one)

- Yes, full time (35 + hours per week)
- Yes, part time
- Yes, but I'm furloughed from my job with pay
- Yes, but I'm furloughed from my job without pay
- No, looking for work
- No, not looking for work (including student or retired)
- Other (please specify) ____________

What is your annual or yearly personal income? (Select one)

- $0-$9,999
- $10,000-$19,999
- $20,000-$29,999
- $30,000-$39,999
- $40,000-$49,999
- $50,000-$59,999
- $60,000-$69,999
- $70,000-$79,999
- $80,000-$89,999
- $90,000-$99,000
- $100,000+
- Prefer not to answer

What is the highest level of education you have completed? (Select one)

- ○ Did not finish high school
- ○ High school diploma or GED
- ○ Some college or associates degree (2-year degree)
- ○ Bachelor's degree (4-year degree)
- ○ Graduate degree

In what zip code do you currently reside? (if you prefer not to answer, please leave the text box blank)

[ ]

How would you describe your political party affiliation? (Select one)

- ○ Democratic
- ○ Republican
- ○ Independent
- ○ [ ] Other

Please indicate which best describes your current living situation. (Select one)

- ○ I live alone
- ○ I live with family
- ○ I live with friends/roommates

Raffle

If you would like to be entered into a raffle for a chance to be randomly selected to win one of five $20 Walmart gift cards, please enter your email address below. If you prefer not to share your email address, or if you would not like to enter the raffle, please leave this space blank.

Raffle Notification

If you entered your email address for the raffle, we will contact you if you are selected. If you have any questions or concerns about the survey, feel free to reach our team at vaxc19a@iu.edu. Thank you again for your time.

Powered by Qualtrics
