# Supplementary material for: COVID-19 vaccine: A 2021 analysis of perceptions on vaccine safety and promise in a U.S. sample
Source: PLoS One. 2022 May 19;17(5):e0268784. doi: 10.1371/journal.pone.0268784 (PMC9119541; doi:10.1371/journal.pone.0268784)
Supplement: S1 Data — (DOCX) [file pone.0268784.s002.docx]

if progress < **90** then delete;

if age_cat = "A: Younger than 18" then delete;

if status = **8** then delete;

if a2 = **.** & a4_1 = **1** then covid_vaccination_received = "A: Yes";

else if a2 = **.** & a4_2 = **1** then covid_vaccination_received = "A: Yes";

else if a2 = **.** & a4_3 = **1** then covid_vaccination_received = "A: Yes";

else if a2 = **.** then delete;

if diagnosed_covid = "" then delete;

if believe_had_covid = "" then delete
